# Antimicrobial susceptibility and clarithromycin resistance patterns of *Helicobacter pylori* clinical isolates in Vietnam

**DOI:** 10.12688/f1000research.8239.1

**Published:** 2016-04-13

**Authors:** Camelia Quek, Son T. Pham, Kieu T. Tran, Binh T. Pham, Loc V. Huynh, Ngan B.L. Luu, Thao K.T. Le, Kelly Quek, Van H. Pham

**Affiliations:** 1 Department of Biochemistry and Molecular Biology, University of Melbourne, Melbourne, Australia; 2 Sydney Medical School, University of Sydney, Sydney, Australia; 3 Department of Thoracic Head/Neck Medical Oncology, University of Texas MD Anderson Cancer Center, Houston, USA; 4 Department of Research and Development, NK-Biotek, Ho Chi Minh, Vietnam; 5 School of Medicine, University of Medicine and Pharmacy, Ho Chi Minh, Vietnam; 6 School of Medicine, Tan Tao University, Duc Hoa, Vietnam

**Keywords:** Helicobacter pylori, antimicrobial resistance, 23S rRNA, mutation, gastric ulcer

## Abstract

*Helicobacter pylori* is a gastric pathogen that causes several gastroduodenal disorders such as peptic ulcer disease and gastric cancer.  Eradication efforts of *H. pylori* are often hampered by antimicrobial resistance in many countries, including Vietnam.  Here, the study aimed to investigate the occurrence of antimicrobial resistance among *H. pylori* clinical isolates across 13 hospitals in Vietnam.  The study further evaluated the clarithromycin resistance patterns of *H. pylori* strains.  In order to address the study interests, antimicrobial susceptibility testing, epsilometer test and PCR-based sequencing were performed on a total of 193 strains isolated from patients, including 136 children (3–15 years of age) and 57 adults (19–69 years of age).  Antimicrobial susceptibility testing showed that the overall resistance to amoxicillin, clarithromycin, levofloxacin, metronidazole, and tetracycline was 10.4%, 85.5%, 24.4%, 37.8%, and 23.8% respectively.  The distribution of minimum inhibitory concentrations (MICs) of clarithromycin-resistant strains was 85.5% with MIC >0.5 μg/mL.  The majority of the clarithromycin resistant isolates (135 of 165 subjects) have MICs ranging from 2 μg/mL to 16 μg/mL.  Furthermore, sequencing detection of mutations in 23S rRNA gene revealed that strains resistant and susceptible to clarithromycin contained both A2143G and T2182C mutations.  Of all isolates, eight clarithromycin-resistant isolates (MIC >0.5 μg/mL) had no mutations in the 23S rRNA gene.  Collectively, these results demonstrated that a proportion of clarithromycin-resistant *H. pylori* strains, which are not related to the 23S rRNA gene mutations, could be potentially related to other mechanisms such as the presence of an efflux pump or polymorphisms in the CYP2C19 gene.  Therefore, the present study suggests that providing susceptibility testing prior to treatment or alternative screening strategies for antimicrobial resistance is important for future clinical practice.  Further studies on clinical guidelines and treatment efficacy are pivotal for successful eradication of *H. pylori* infection.

Keywords: *Helicobacter pylori*, antimicrobial resistance, 23S rRNA, mutation, gastric ulcer

## Introduction

*Helicobacter pylori* is a Gram-negative bacterium that plays a causative role in the development of gastric adenocarcinoma, peptic ulcer disease and chronic gastritis ^1, 2^. The prevalence of *H. pylori* infection is more than half of the world’s population, comprising of >80% in developing countries and approximately 40% in the United States ^3, 4^. In Vietnam, the prevalence of *H. pylori* is approximately 80% in adults and 26%–71.4% in children ^5– 7^.

Eradication therapy of symptomatic *H. pylori* infection substantially prevents the recurrence and reduces the risk of developing gastroduodenal-associated diseases ^8– 11^. Recommended therapy, triple-therapy regimen, composed of two antimicrobial agents (e.g. amoxicillin, metronidazole, tetracycline, levofloxacin, and clarithromycin) in combination with a proton pump inhibitor (PPI), has been widely used to eliminate the bacteria ^12– 14^. However, *H. pylori* antimicrobial resistance is increasing worldwide, contributing to the main factor that affects the efficacy of current therapeutic regimens ^15, 16^. Resistance to clarithromycin is believed to be the main factor in treatment failure ^16, 17^. In Vietnam, many studies showed that *H. pylori* is highly resistant to clarithromycin; 33%–34% primary and 74% secondary resistance ^18– 20^. The majority of clarithromycin-resistant strains are identified based on point mutations in the peptidyltransferase region of domain V of 23S rRNA, which affects the binding of macrolides to the bacterial ribosome ^21– 23^.

The common 23S rRNA point mutations (e.g. A2143G, A2142C/G and T2182C) are recommended for rapid routine diagnostic procedures, as compared to the time-consuming bacterial culture. A plethora of studies have evidently reported the association of minimum inhibitory concentrations (MICs) of clarithromycin-resistance strains to the respective point mutation ^24– 27^. For example, A2142C/G mutations are associated with MIC >256 μg/mL, and mutations such as A2143G and T2182C are associated with MIC >0.5 μg/mL ^27, 28^. However, it is unclear whether such association between point mutation and MIC can be utilised as predictors for strains resistant to clarithromycin ^23, 29– 31^. Here, the present study evaluated the antimicrobial resistance of *H. pylori* strains isolated from patients in Vietnam with the following antimicrobial agents: amoxicillin, metronidazole, tetracycline, levofloxacin and clarithromycin. The strains resistant to clarithromycin were further investigated to assess the point mutations in the 23S rRNA gene and MIC values as predictors for screening *H. pylori* strains. The overall findings addressed the issues of using 23S rRNA mutations in clinical diagnosis.

## Materials and methods

### Study samples

The present work was designed as a prospective randomised clinical study across 13 hospitals (Children's Hospital 2, Children's Hospital 1, Trieu An Hospital, Tam Nhat Clinic, Dai Phuoc Clinic, Hoan My Hospital, DH Y Duoc Hospital, Phap Viet Hospital, Yersin International Clinic, Dong Nai International Hospital, Nguyen Tri Phuong Hospital, Van Hanh General Hospital, Gia Dinh People's Hospital) in Ho Chi Minh City, Vietnam, from July 2015 to January 2016 (Data availability). The study was approved by Nam Khoa Biotek Diagnostic Ethics Committee (ID: NCKH 04/02-15/NK). Written informed consent was obtained from each patient or the patient’s parents for the use of this study. Biopsy specimens of the gastric mucosa were obtained from 193 patients, including 136 children (3–15 years of age) and 57 adults (19–69 years of age). These patients showed indication of endoscopy for the examination of dyspeptic symptoms (i.e. gastric ulcer).

### *Helicobacter pylori* culture and antimicrobial susceptibility testing

The *H. pylori* culture and susceptibility testing were performed as described in previous studies ^32, 33^. Briefly, biopsy specimens were homogenised in 500 µL transport medium (20% glycerol; 0.9% NaCl in Milli-Q water), and were subsequently inoculated onto *H. pylori* selective agar plates at 37°C in a microaerophilic atmosphere. Biochemical identification of *H. pylori* was performed using Gram stain (Gram negative), oxidase test (oxidase positive), catalase test (catalase positive) and urease test (urease positive). Susceptibility testing was performed on Muller-Hinton agar plates supplemented with 10% lysed horse blood for the following antibiotics: amoxicillin (0.25 μg/mL), clarithromycin (0.75 μg/mL), levofloxacin (1 μg/mL), metronidazole (8 μg/ml), and tetracycline (2 μg/mL). The MIC values were obtained by the epsilometer test (E-test; bioMerieux, Marcy I’Etoile, France) for clarithromycin in accordance with the manufacturer’s protocol using 10% lysed horse blood supplemented in Mueller-Hinton Z agar (bioMerieux). Bacterial suspensions were prepared in Mueller-Hinton broth and adjusted to a McFarland turbidity of three. Resistance criteria for clarithromycin was defined according to the European Committee on Antimicrobial Susceptibility Testing (EUCAST); susceptible (MIC ≤0.25 μg/mL) and resistance (MIC >0.5 μg/mL) ^34^.

### PCR amplification and sequence detection of 23S rRNA mutation

The PCR mixture (20-µL final volume) contained HotStar Taq master mix (Qiagen, Hilden, Germany) and 10 pmol of forward DP1 (5’- **GTAAAACGACGGCCAGT** ACGGCGGCCGTAACTATA-3’) and reverse ZGE23 (5’- **TATTTAGGTGACACTATAG** ACAGGCCAGTTAGCTA-3’) primers. These primers contain sequences (written in bold-faced type) that are specific for SP6 (DP1) and M13 (ZGE23), and underlined sequences indicate 23S rRNA amplicon of 308 bp comprising of 2142, 2143 and 2182 positions. *H. pylori* colonies on selective medium was added to 1× TE buffer (10 mM Tris-HCL, 1 mM EDTA, pH 7.6) and heated up to 100 ^°^C for 5 min, followed by centrifugation at 8000 rpm. 1 µL of supernatant was added to the PCR mix to amplify 23S rRNA gene. The PCR cycling conditions were 95 ^°^C for 15 min to activate HotStart Taq DNA polymerase, followed by 40 cycles of 94 ^°^C for 15 sec, 57 ^°^C for 30 sec, 72 ^°^C for 30 sec, and final extension at 72 ^°^C for 5 min. The PCR products were purified prior to sequencing by Illustra ExoStar 1-Step (GE Healthcare Life Sciences, Buckinghamshire, United Kingdom) according to manufacturer’s instructions, and followed by Big-Dye (Perkin-Elmer Applied Biosystems, Foster City, USA) amplification using SP6 and M13 primers. Sequencing was then performed using ABI 3130XL sequencer. In total, 193 sequences were obtained and analysed using MEGA version 5.0 ^35^ against wild-type 23S rRNA gene available in the GenBank ^36^ database (Accession number: U27270). Sequence data can be downloaded from GenBank database (Accession numbers: KU904824-KU905015).

### Statistical analysis

Mann-Whitney *t*-test, unpaired two-tailed was used to compare resistance rate between different patient groups. All analyses were performed using SPSS Statistics version 20 (SPSS, Chicago, USA) and Prism version 5.0 (GraphPad, San Diego, USA).

## Results

A summary of patient information, antimicrobial susceptibility and clarithromycin resistance patterns The table summarised individual patient records such as age, gender, hospital, and presence of *H. pylori* strains. Antimicrobial susceptibility testing and clarithromycin resistance patterns of each patient is also included in the data. Click here for additional data file. Copyright: © 2016 Quek C et al. 2016 Data associated with the article are available under the terms of the Creative Commons Zero "No rights reserved" data waiver (CC0 1.0 Public domain dedication).

### Antimicrobial resistance of *Helicobacter pylori* isolates

**Figure 1.**
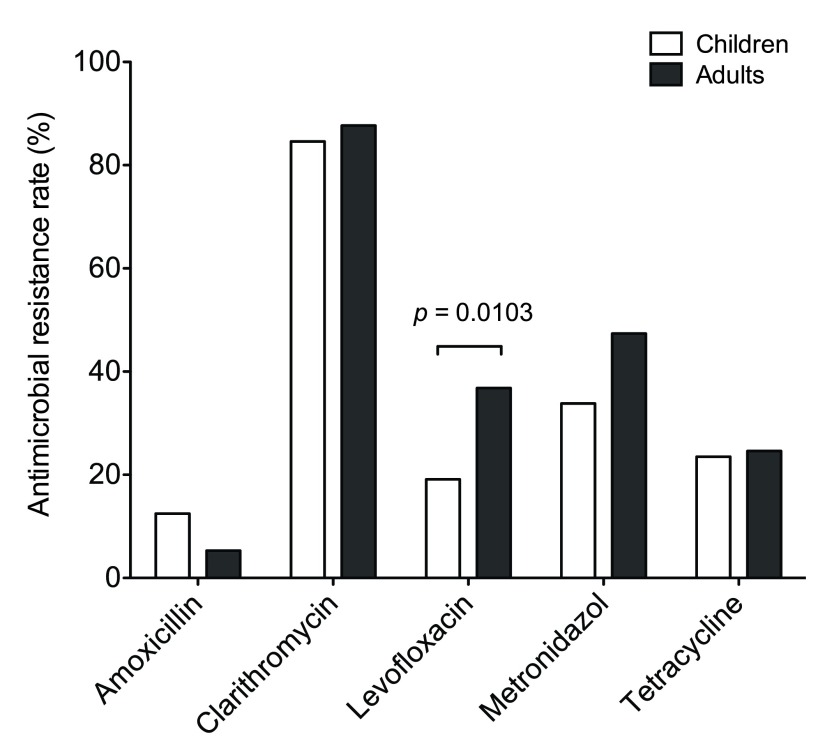
Antimicrobial resistance rate of *Helicobacter pylori* isolates from Vietnamese children and adults. The graph displays the resistance rate of amoxicillin, clarithromycin, levofloxacin, metronidazole, and tetracycline in both children and adults. Among the antimicrobial agents, clinical isolates resistant to levofloxacin is significantly higher ( *p* = 0.0103) in adults than in children.

To assess the antimicrobial resistance of *H. pylori* in Vietnam, susceptibility testing was performed and the resistance rate of each antimicrobial is listed in Table 1. The prevalence of antimicrobial resistance was detected in the following order, from highest to lowest: clarithromycin, metronidazole, levofloxacin, tetracycline and amoxicillin. Of all the antimicrobial agents, the majority of isolates were resistant to clarithromycin as shown in 85.5% of all patients (84.6% in children and 87.7% in adults). The occurrence of metronidazole resistance was lower than clarithromycin (overall 37.8% vs. 85.5%) in this study, as compared to the other published reports ^18– 20^. Antimicrobial resistance in adults is predominately higher than children, except for amoxicillin resistance which occurred in 12.5% of children and 5.3% of adults without statistical significance. A statistically significant difference was observed in the resistance rate of levofloxacin ( *p* = 0.0103) between children and adults in Figure 1.

**Table 1.** Prevalence of antimicrobial resistance in *Helicobacter pylori* isolates.

Antimicrobial resistance	Children, *n* = 136 N (%)	Adults, *n* = 57 N (%)	Total, *n* = 193 N (%)
Amoxicillin	17 (12.5)	3 (5.3)	20 (10.4)
Clarithromycin	115 (84.6)	50 (87.7)	165 (85.5)
Levofloxacin	26 (19.1)	21 (36.8)	47 (24.4)
Metronidazole	46 (33.8)	27 (47.4)	73 (37.8)
Tetracycline	32 (23.5)	14 (24.6)	46 (23.8)

### Minimum inhibitory concentration values of clarithromycin-resistant isolates predominately range from 2 μg/ml to 16 μg/ml

**Figure 2.**
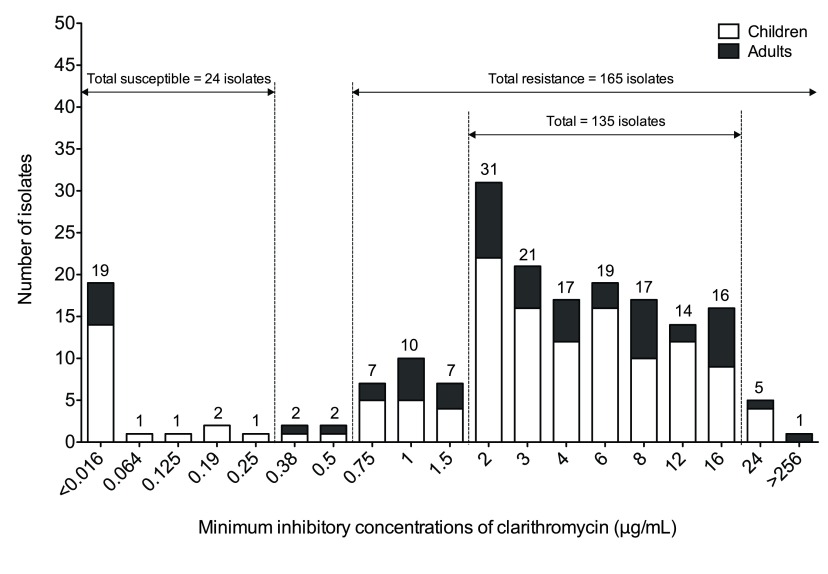
Minimum inhibitory concentration values of clarithromycin susceptible and resistant isolates in children and adults. The graph shows the number of isolates across a range of minimum inhibitory concentration values of clarithromycin. The total number of clarithromycin susceptible and resistant isolates is 24 and 165, respectively. Majority of clinical isolates resistant to clarithromycin have MIC values ranging from 2 μg/mL to 16 μg/mL.

To validate the clarithromycin resistant isolates, MIC values were obtained from a total of 193 clinical isolates using an E-test. Based on EUCAST proposed breakpoints, the respective occurrence of clarithromycin susceptible and resistant isolates was 24 (12.4%) and 165 (85.5%) of the total number of isolates used in this study. The distribution of MICs showed that the majority of clinical isolates resistant to clarithromycin (135 of 165 isolates, 81.8%, including 97 children and 38 adults) ranged from 2 μg/mL to 16 μg/mL ( Figure 2). Of all isolates, only five subjects (including four children and one adult) showed a MIC of 24 μg/mL, and one adult subject had a MIC >256 μg/mL ( Figure 2).

### Mutations of 23S rRNA gene in *Helicobacter pylori* isolates

To investigate the point mutations in the 23S rRNA gene of clarithromycin-resistant isolates, mutations at position 2142 (A2142G or A2142C), 2143 (A2143G), and 2182 (T2182C) were analysed in this study. Sequence analyses showed the point mutations in the 23S rRNA gene were detected not only in clarithromycin-resistant isolates, but also in clarithromycin-susceptible isolates. In Table 2, both A2143G and T2182C mutations were predominantly detected in 91.7% ( *n* = 177) of the clarithromycin-susceptible and –resistant isolates. Only two clarithromycin-resistant isolates in adults had the A2142G and T2182C mutations with a respective MIC value of 8 µg/mL and >256 µg/mL. In addition, a total of 10 clarithromycin-resistant and –susceptible isolates had no mutations in the 23S rRNA gene. The present study also identified four isolates with both A2143G and T2182C mutations at MIC values ranging from 0.38 to 0.5 μg/mL, which are considered to be intermediate resistance strains ^34^.

**Table 2.** Minimum inhibitory concentration values and 23S rRNA mutations of clarithromycin-susceptible and -resistant isolates.

Mutation(s)	No. of susceptible isolates		No. of resistance isolates		Total N (%)	MICs (μg/mL)
	Children	Adults	Children	Adults		
A2143G + T2182C	19	3	112	43	177 (91.7)	≤0.25 (S) and >0.5 (R)
A2142G + T2182C	0	0	0	2	2 (1.0)	8 and >256
A2143G + T2182C*	N.A.	N.A.	N.A.	N.A.	4 (2.1)	0.38 and 0.5
No mutations	0	2	3	5	10 (5.2)	>0.016 and ≤12
Total					193 (100)

‘*’ indicates *H. pylori* isolates with A2143G and T2182C mutation at MIC values, which are considered to be intermediate resistance strains. Abbreviations: ‘N.A.’ – not applicable; ‘S’ – susceptible; ‘R’ – resistance.

## Discussion

Antimicrobial resistance in *H. pylori* has become a global health problem because the prevalence of infection and incidence is increasing worldwide ^37– 39^. The increasing *H. pylori* resistance to antimicrobial agents, such as clarithromycin, is considered the main factor for reduced treatment success in several countries, including Vietnam and Japan ^17, 18, 40– 42^. Therefore, the understanding of geographical region specific prevalence is crucial for treatment of *H. pylori* infection.

Vietnam is categorised as a region with a high prevalence of *H. pylori* infection and an intermediate risk of gastric cancer ^43, 44^. In Vietnam (Ho Chi Minh City and Hanoi), clarithromycin and metronidazole are recommended as a first-line therapy regimen ^13^. Our present study showed that the overall resistance rate for clarithromycin and metronidazole was 85.5% and 37.8%, respectively. The high incidence of *H. pylori* strains resistant to clarithromycin and metronidazole in Vietnam might be attributed to the following: (i) unregulated or widespread over-the-counter use of antibiotics, (ii) clarithromycin is prescribed frequently for treatment due to its high bactericidal effect, and (iii) antibiotics are often used to treat *H. pylori* infection and other infections including respiratory tract infections (clarithromycin) and intestinal parasites (metronidazole) ^18, 45, 46^. Of note, this study highlighted that clarithromycin resistance was the highest among the 193 *H. pylori* clinical isolates collected in 2015−2016, as compared to the other studies in which metronidazole has the highest resistance rate (69.9%−76.1%) in Vietnam ^18– 20^. The observation of high clarithromycin resistance rate from our data suggested the increasing occurrence of resistant strains among other antimicrobial agents. Therefore, constant surveillance for antimicrobial resistance rates is necessary to gain insights into effective eradication therapy of *H. pylori* infection.

Another interest of this study was to assess the variations of MIC values obtained from the clarithromycin-resistant strains. Our representative clinical isolates obtained from the gastric mucosa revealed that the majority of strains resistant to clarithromycin conferred MIC values ranging from 2 μg/mL to 16 μg/mL. There is also a degree of variation on the MIC range between studies ^19, 32, 47– 49^. The variability of MIC values for resistant isolates might be attributed to different gastric sites. The evidence is supported by Borody *et al.* who demonstrated that the bimodal distribution of clarithromycin resistance of isolates cultured from 4 gastric sites (i.e. antrum, distal body, proximal body and fundus) ranged from <0.016 μg/mL to 256 μg/mL ^50^. The recent studies also demonstrated that MIC values for clarithromycin resistance vary at different gastric sites ^47– 49^. Therefore, the present results confirm previous studies that multiple gastric biopsies from different sites of the stomach are crucial for accurate diagnosis of *H. pylori* infection.

Furthermore, antimicrobial susceptibility testing using MIC values is often used to determine the appropriate dosage of antimicrobial for a patient’s prescription. However, the respective antimicrobial resistance rate is based on the defined MIC breakpoints, which are much lower than the achievable tissue concentrations of antimicrobial agents such as clarithromycin (ranging from 5.2 μg/mL to 22.2 μg/mL) ^51^. Only a few studies have reported the eradication rate of *H. pylori* infection with high MIC values (e.g. >24 μg/mL), highlighting that the significant eradication rate of 50%−80% on MIC-defined resistant strains can be achieved by administering PPI with precise antibiotic dosage and appropriate treatment duration ^20, 32, 52, 53^. Hence, further longitudinal studies on treatment efficacy and treatment guidelines are necessary for successful treatment.

Point mutations at positions 2142, 2143 and 2182 on the 23S rRNA gene were commonly reported ^25– 27^. Yet it remains unclear whether or not these point mutations could be a strong predictor of clarithromycin resistance ^23, 29– 31^. In some studies, only the A2142G mutation was found to be associated with high MIC values ^54, 55^. While other studies showed that mutations at positions 2142 and/or 2143 were associated with clarithromycin resistance ^53, 54, 56, 57^. In addition, mutation T2182C was only reported in one study ^25^. Here, we reported that *H. pylori* strains with mutations in A2143G and T2182C exhibited not only in clarithromycin-resistant strains, but also in susceptible strains as observed in Table 2. Similar to Phan *et al.*’s study ^19^, none of the clarithromycin-resistant strains portrayed A2142C mutation in our study. It is important to note that the association of MIC values and point mutations was not identified in our work. Additionally, a proportion of all isolates had no point mutations in the 23S rRNA gene ( Table 2). Further investigation on other nucleotide positions of the 23S rRNA region should be performed on these resistant strains ^58, 59^. Additionally, we suggested that a proportion of these resistant strains, which are not related to the 23S rRNA gene sequence, could be potentially related to other mechanisms such as the presence of an efflux pump (e.g. outer membrane protein hefA) or polymorphisms in the CYP2C19 gene ^60– 62^.

## Conclusions

In conclusion, our present results confirm that MIC values are critical for accurate identification of antimicrobial resistant strains. Susceptibility tests prior to treatment are necessary to select the optimal *H. pylori* therapy regimens in Vietnam. Further studies on other resistance mechanisms, particularly the mutations of the host genes, will provide additional insights into the development of diagnostic biomarkers and therapeutic drugs.

## Consent

Written informed consent for publication of their clinical details was obtained from the parents of the patients.

## Data availability

The data referenced by this article are under copyright with the following copyright statement: Copyright: © 2016 Quek C et al.

Data associated with the article are available under the terms of the Creative Commons Zero "No rights reserved" data waiver (CC0 1.0 Public domain dedication). 

*F1000Research*: Dataset 1. A summary of patient information, antimicrobial susceptibility and clarithromycin resistance patterns, 10.5256/f1000research.8239.d118249 ^63^
