# Supplementary material for: Antimicrobial susceptibility and clarithromycin resistance patterns of Helicobacter pylori clinical isolates in Vietnam
Source: F1000Res. 2016 Apr 13;5:671. [Version 1] doi: 10.12688/f1000research.8239.1 (PMC4972085; doi:10.12688/f1000research.8239.1)
Supplement: Peer review history

# Peer review: Referee response for version 1

**DOI:** 10.5256/f1000research.8861.r13392

**Published:** 2016-08-02

**Authors:** Simon Cutting

**Affiliations:** 1 School of Biological Sciences, Royal Holloway, University of London, Egham, UK

In general this is an interesting paper reporting on resistance ot the antibiotic clarithromycin in Vietnam and the relevance of this resistance to Helicobacter pylori infection. The main outcome of this work is that it demonstrates the need for susceptibility testing prior to treatment. It is encouraging that this work is led by Vietnamese scientists and the work appears of a high standard.

 It would have been useful to have a figure showing the mutational hotspots within the 23S rRNA gene.

**General**

 Bacterial species names should be written in italics

 The MICs stated on page 3 (3 ^rd^ para) show considerable variation with a large range, i.e., >256 mg/ml and >0.5 mg/ml. What is considered significant?

 MIC should be used as an abbreviation in Figures and Tables as well as text, e.g. Fig 2 and Table 2

I have read this submission. I believe that I have an appropriate level of expertise to confirm that it is of an acceptable scientific standard.

# Peer review: Referee response for version 1

**DOI:** 10.5256/f1000research.8861.r14071

**Published:** 2016-05-31

**Authors:** Duc Trong Quach

**Affiliations:** 1 Department of Internal Medicine, University of Medicine and Pharmacy, Ho Chi Minh City, Vietnam

This is one among the informative studies about the antimicrobial susceptibility and clarithromycin resistance patterns of Helicobacter pylori clinical isolates in Vietnam. The title is appropriate for the content. The methods and analysis of the results are well-described and appropriate.

 However, there are several minor revisions that the authors should be considered to make results of the paper more clinically meaningful:

1. The design of this study is not prospective randomized one. It is a cross-sectional study.
2. The clinical information of the patients recruited in the study should be clarified: are they naïve patients or not. This information is essential to understand the true situation of antimicrobial susceptibility in Vietnam. As a result, the conclusion “Susceptibility tests prior to treatment are necessary to select the optimal *H. pylori* therapy regimens in Vietnam” may be not appropriate without this information.
3. The authors should also addressed the weak points of the studies. Although this is a multi-center study, all of the medical centers locates in southern Vietnam. The picture of antimicrobial susceptibility and clarithromycin resistance patterns of Helicobacter pylori has been shown to be somewhat different in Central and Northern Vietnam. Therefore, the author should change the title from “in Vietnam” to “in southern Vietnam”, or they can keep the title as it was but add a sentence which mentions this weak point of the study.

I have read this submission. I believe that I have an appropriate level of expertise to confirm that it is of an acceptable scientific standard.
